# 1-(4a,8-Dimethyl-1,2,3,4,4a,5,6,8a-octa­hydro­naphthalen-2-yl)-3-phenyl­prop-2-en-1-one

**DOI:** 10.1107/S1600536811010452

**Published:** 2011-03-26

**Authors:** Mohamed Tebbaa, Ahmed Benharref, Moha Berraho, Jean-Claude Daran, Mohamed Akssira, Ahmed Elhakmaoui

**Affiliations:** a Laboratoire de Chimie Bioorganique et Analytique, URAC 22. BP 146, FSTM, Université Hassan II, Mohammedia-Casablanca 20810 Mohammedia, Morocco; b Laboratoire de Chimie Biomoleculaire, Substances Naturelles et Réactivite, URAC16, Université Cadi Ayyad, Faculté des Sciences Semlalia, BP 2390, Bd My Abdellah, 40000 Marrakech, Morocco; c Laboratoire de Chimie de Coordination, 205 route de Narbonne, 31077 Toulouse Cedex 04, France

## Abstract

The title compound, C_21_H_26_O, was semisynthesized from isocostic acid, isolated from the aerial part of *Inula Viscosa­* (L) Aiton [or *Dittrichia Viscosa­* (L) Greuter]. The cyclo­hexene ring has a half-chair conformation, whereas the cyclo­hexane ring displays a chair conformation.

## Related literature

For background to the medicinal inter­est in *Inula Viscosa­* (L) Aiton [or *Dittrichia Viscosa­* (L) Greuter], see: Shtacher & Kasshman (1970 ▶); Bohlman & Gupta (1982 ▶); Azoulay *et al.* (1986 ▶); Bohlmann *et al.* (1977 ▶); Ceccherelli *et al.* (1988 ▶). For the synthesis, see: Kutney & Singh (1984 ▶). For conformational analysis, see: Cremer & Pople (1975 ▶).
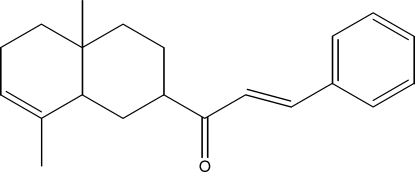


## Experimental

### 

#### Crystal data

C_21_H_26_O
*M* *_r_* = 294.42
Orthorhombic, 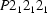

*a* = 9.5760 (8) Å
*b* = 11.3542 (11) Å
*c* = 15.7852 (13) Å
*V* = 1716.3 (3) Å^3^
*Z* = 4
Mo *K*α radiation
μ = 0.07 mm^−1^
*T* = 180 K
0.37 × 0.16 × 0.16 mm

#### Data collection

Agilent Xcalibur Eos Gemini ultra diffractometer
Absorption correction: multi-scan (*CrysAlis PRO*; Agilent, 2010 ▶) *T*_min_ = 0.817, *T*_max_ = 1.000
10251 measured reflections
3436 independent reflections
3180 reflections with *I* > 2σ(*I*)
*R*_int_ = 0.022

#### Refinement

*R*[*F* ^2^ > 2σ(*F* ^2^)] = 0.037
*wR*(*F* ^2^) = 0.089
*S* = 1.05
3436 reflections
201 parameters
H-atom parameters constrained
Δρ_max_ = 0.15 e Å^−3^
Δρ_min_ = −0.15 e Å^−3^

### 

Data collection: *CrysAlis PRO* (Agilent, 2010 ▶); cell refinement: *CrysAlis PRO*; data reduction: *CrysAlis PRO*; program(s) used to solve structure: *SHELXS97* (Sheldrick, 2008 ▶); program(s) used to refine structure: *SHELXL97* (Sheldrick, 2008 ▶); molecular graphics: *ORTEP-3 for Windows* (Farrugia, 1997 ▶); software used to prepare material for publication: *WinGX* (Farrugia, 1999 ▶).

## Supplementary Material

Crystal structure: contains datablocks I, global. DOI: 10.1107/S1600536811010452/fj2408sup1.cif

Structure factors: contains datablocks I. DOI: 10.1107/S1600536811010452/fj2408Isup2.hkl

Additional supplementary materials:  crystallographic information; 3D view; checkCIF report

## Figures and Tables

**Fig. 1.**
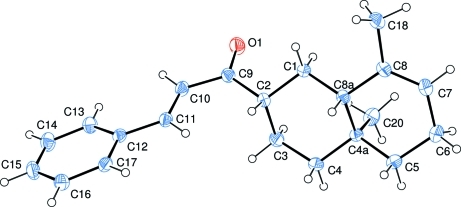
: Molecular structure of the title compound with the atom-labelling scheme. Displacement ellipsoids are drawn at the 30% probability. level. H atoms are represented as small spheres of arbitrary radii.

## supplementary crystallographic information

### Comment

Our work lies within the framework of the valorization of medicinals plants and concerning Inula Viscosa(*L*) Aiton or Dittrichia Viscosa (*L*) Greuter. This plant is widespread in Mediterranean area and extends to the Atlantic cost of Morocco. It is a well known medicinal plant (Shtacher & Kasshman, 1970; Bohlman & Gupta, 1982) and has some pharmacological activities (Azoulay *et al.*, 1986). This plant has been the subject of chemical investigation in terms of isolating sesquiterpene lactones (Bohlmann *et al.*, 1977), sesquiterpene acids (Ceccherelli *et al.*, 1988). The isocostic acid is a major constituent of the dichloromethane extract of the Inula viscosa (*L*).The literature does not report any article on the transformation of this acid. In order to prepare products with high added value, we studied the reactivity of this acid. Thus, from this acid, we have prepared by reaction of Curtius the 1 - (4a, 8dimethyl-1,2,3,4,4a,5,6,8a-octahydronaphthalen-2-yl)- ethanone which was synthesized (Kutney *et al.*,1984). The Condensation of this ketone with benzaldehyde in the presence of sodium hydroxide allows us to obtain the title compound with a good yield of 85%. The structure of this new derivative of isocostic acid was established by NMR spectral analysis of 1H, 13 C and mass spectroscopy and confirmed by its single-crystal X-ray structure. The molecule is built up from two fused six-membered rings, substituted by 3-phenylpropenoyl. The molecular structure of (I), Fig.1, shows the cyclohexane ring to adopt a chair conformation, as indicated by the total puckering amplitude QT = 0.5674 (19)Å and spherical polar angle θ =4.83 (19)° with φ = 266 (2)°. While the cyclohexene ring has a half chair conformation with QT = 0.5005 (19) Å, θ =47.9 (2)°, φ = 13.7 (3)° (Cremer & Pople, 1975).

### Experimental

In a flask was introduced a mixture of 500 mg (2.42 mmol), of 1 - (4a, 8-dimethyl-1, 2,3,4,4a,5,6,8a-octahydronaphthalen- 2-yl)-ethanone, 257 mg (2.42 mmol.) of benzaldehyde, 30 ml of anhydrous ethanol and 1 ml of a solution of sodium hydroxide(2 N). The mixture was stirred for three hours at room temperature. After neutralization followed by extraction three time with 20 ml of dichloromethane, the organic phase is dried over sodium sulfate, then evaporated under vacuum. Chromatography on a column of silica gel with hexane-ethyl acetate (97/3) as eluent of the residue allowed us to obtain 1-(4a, 8-dimethyl-1,2,3,4,4a, 5,6,8a-octahydronaphthalen-2-yl)-3-phenylprop-2-en-1-one with a yield of 85%. The title compound is recrystallized in hexane- ethyl acetate (80/20).

### Refinement

All H atoms were fixed geometrically and treated as riding with C—H = 0.96 Å (methyl), 0.97 Å (methylene), 0.98Å (methine) with *U*_iso_(H) = 1.2U_eq_ (methylene, methine) or *U*_iso_(H) = 1.5*U*_eq_ (methyl). In the absence of significant anomalous scattering, the absolute configuration could not be reliably determined and thus 1436 Friedel pairs were merged and any references to the Flack parameter were removed.

### Crystal data

**Table:**

C_21_H_26_O	*F*(000) = 640
*M**_r_* = 294.42	*D*_x_ = 1.139 Mg m^−^^3^
Orthorhombic, *P*2_1_2_1_2_1_	Mo *K*α radiation, λ = 0.71073 Å
Hall symbol: P 2ac 2ab	Cell parameters from 6842 reflections
*a* = 9.5760 (8) Å	θ = 3.3–27.2°
*b* = 11.3542 (11) Å	µ = 0.07 mm^−^^1^
*c* = 15.7852 (13) Å	*T* = 180 K
*V* = 1716.3 (3) Å^3^	Box, colorless
*Z* = 4	0.37 × 0.16 × 0.16 mm

### Data collection

**Table:**

Agilent Xcalibur Eos Gemini ultra diffractometer	3436 independent reflections
Radiation source: Enhance (Mo) X-ray Source	3180 reflections with *I* > 2σ(*I*)
graphite	*R*_int_ = 0.022
Detector resolution: 16.1978 pixels mm^-1^	θ_max_ = 26.4°, θ_min_ = 3.3°
ω scans	*h* = −11→11
Absorption correction: multi-scan (*CrysAlis PRO*; Agilent, 2010)	*k* = −14→14
*T*_min_ = 0.817, *T*_max_ = 1.000	*l* = −19→19
10251 measured reflections	

### Refinement

**Table:**

Refinement on *F*^2^	Primary atom site location: structure-invariant direct methods
Least-squares matrix: full	Secondary atom site location: difference Fourier map
*R*[*F*^2^ > 2σ(*F*^2^)] = 0.037	Hydrogen site location: inferred from neighbouring sites
*wR*(*F*^2^) = 0.089	H-atom parameters constrained
*S* = 1.05	*w* = 1/[σ^2^(*F*_o_^2^) + (0.0379*P*)^2^ + 0.290*P*] where *P* = (*F*_o_^2^ + 2*F*_c_^2^)/3
3436 reflections	(Δ/σ)_max_ < 0.001
201 parameters	Δρ_max_ = 0.15 e Å^−^^3^
0 restraints	Δρ_min_ = −0.15 e Å^−^^3^

### Special details

**Table:**

Experimental. Empirical absorption correction using spherical harmonics, implemented in SCALE3 ABSPACK scaling algorithm. CrysAlisPro (Agilent Technologies, 2010)
Geometry. All s.u.'s (except the s.u. in the dihedral angle between two l.s. planes) are estimated using the full covariance matrix. The cell s.u.'s are taken into account individually in the estimation of s.u.'s in distances, angles and torsion angles; correlations between s.u.'s in cell parameters are only used when they are defined by crystal symmetry. An approximate (isotropic) treatment of cell s.u.'s is used for estimating s.u.'s involving l.s. planes.
Refinement. Refinement of *F*^2^ against ALL reflections. The weighted *R*-factor *wR* and goodness of fit *S* are based on *F*^2^, conventional *R*-factors *R* are based on *F*, with *F* set to zero for negative *F*^2^. The threshold expression of *F*^2^ > σ(*F*^2^) is used only for calculating *R*-factors(gt) *etc*. and is not relevant to the choice of reflections for refinement. *R*-factors based on *F*^2^ are statistically about twice as large as those based on *F*, and *R*- factors based on ALL data will be even larger.

### Fractional atomic coordinates and isotropic or equivalent isotropic displacement parameters (Å^2^)

**Table:**

	*x*	*y*	*z*	*U*_iso_*/*U*_eq_	
C8A	0.19916 (15)	0.40195 (13)	0.37785 (8)	0.0301 (3)	
H1	0.2470	0.4777	0.3713	0.036*	
C8	0.13546 (16)	0.40428 (13)	0.46570 (9)	0.0336 (3)	
C7	0.00699 (18)	0.44778 (15)	0.47799 (10)	0.0451 (4)	
H7	−0.0253	0.4519	0.5335	0.054*	
C6	−0.08850 (18)	0.49016 (17)	0.40973 (10)	0.0472 (4)	
H6A	−0.1260	0.5664	0.4256	0.057*	
H6B	−0.1661	0.4358	0.4043	0.057*	
C5	−0.01461 (17)	0.50089 (15)	0.32443 (9)	0.0401 (4)	
H5A	0.0366	0.5746	0.3228	0.048*	
H5B	−0.0840	0.5029	0.2797	0.048*	
C4A	0.08624 (15)	0.39923 (13)	0.30807 (9)	0.0304 (3)	
C4	0.15939 (18)	0.41743 (16)	0.22242 (9)	0.0426 (4)	
H4B	0.0903	0.4132	0.1776	0.051*	
H4A	0.2002	0.4956	0.2212	0.051*	
C3	0.27356 (17)	0.32686 (17)	0.20516 (9)	0.0437 (4)	
H3A	0.3206	0.3466	0.1526	0.052*	
H3B	0.2314	0.2498	0.1983	0.052*	
C2	0.38162 (15)	0.32205 (13)	0.27753 (9)	0.0314 (3)	
H2	0.4344	0.3960	0.2776	0.038*	
C1	0.31110 (15)	0.30807 (13)	0.36395 (8)	0.0313 (3)	
H1B	0.2688	0.2306	0.3676	0.038*	
H1A	0.3809	0.3141	0.4083	0.038*	
C9	0.48216 (15)	0.22168 (13)	0.25939 (9)	0.0332 (3)	
C10	0.60358 (16)	0.24005 (13)	0.20307 (9)	0.0348 (3)	
H10	0.6567	0.1742	0.1894	0.042*	
C11	0.64329 (14)	0.34209 (13)	0.17044 (9)	0.0321 (3)	
H11	0.5932	0.4083	0.1871	0.039*	
C12	0.75915 (15)	0.36131 (13)	0.11029 (8)	0.0309 (3)	
C13	0.82643 (17)	0.27048 (15)	0.06720 (10)	0.0391 (4)	
H13	0.8003	0.1928	0.0769	0.047*	
C14	0.93184 (17)	0.29534 (16)	0.01017 (11)	0.0463 (4)	
H14	0.9767	0.2342	−0.0180	0.056*	
C15	0.97102 (17)	0.41042 (17)	−0.00528 (11)	0.0471 (4)	
H15	1.0415	0.4268	−0.0440	0.056*	
C16	0.90527 (16)	0.50077 (16)	0.03690 (10)	0.0422 (4)	
H16	0.9315	0.5784	0.0268	0.051*	
C17	0.80018 (15)	0.47621 (14)	0.09437 (9)	0.0343 (3)	
H17	0.7564	0.5378	0.1227	0.041*	
C20	0.00546 (18)	0.28311 (15)	0.30817 (11)	0.0445 (4)	
H20A	−0.0312	0.2687	0.3638	0.067*	
H20B	0.0669	0.2200	0.2925	0.067*	
H20C	−0.0701	0.2877	0.2683	0.067*	
C18	0.22086 (18)	0.36189 (18)	0.53939 (9)	0.0472 (4)	
H18C	0.1687	0.3720	0.5908	0.071*	
H18A	0.3058	0.4065	0.5426	0.071*	
H18B	0.2427	0.2800	0.5319	0.071*	
O1	0.46109 (12)	0.12334 (9)	0.28773 (7)	0.0471 (3)	

### Atomic displacement parameters (Å^2^)

**Table:**

	*U*^11^	*U*^22^	*U*^33^	*U*^12^	*U*^13^	*U*^23^
C8A	0.0304 (7)	0.0322 (7)	0.0277 (7)	−0.0013 (6)	0.0012 (6)	−0.0015 (6)
C8	0.0352 (8)	0.0357 (8)	0.0298 (7)	−0.0045 (7)	0.0025 (6)	−0.0017 (6)
C7	0.0428 (9)	0.0567 (10)	0.0358 (8)	0.0089 (8)	0.0101 (7)	0.0016 (7)
C6	0.0403 (9)	0.0534 (10)	0.0480 (9)	0.0150 (8)	0.0076 (7)	0.0009 (8)
C5	0.0392 (8)	0.0421 (8)	0.0389 (8)	0.0102 (8)	−0.0017 (7)	0.0046 (7)
C4A	0.0282 (7)	0.0349 (8)	0.0280 (7)	0.0027 (6)	−0.0013 (6)	0.0010 (6)
C4	0.0414 (9)	0.0582 (10)	0.0283 (7)	0.0103 (8)	−0.0008 (6)	0.0086 (7)
C3	0.0424 (9)	0.0620 (11)	0.0266 (7)	0.0095 (8)	0.0022 (7)	0.0010 (7)
C2	0.0293 (7)	0.0345 (8)	0.0305 (7)	−0.0002 (6)	0.0044 (6)	0.0019 (6)
C1	0.0300 (7)	0.0363 (8)	0.0275 (7)	0.0027 (7)	0.0001 (6)	0.0024 (6)
C9	0.0319 (7)	0.0373 (8)	0.0305 (7)	−0.0004 (7)	0.0008 (6)	−0.0010 (7)
C10	0.0328 (8)	0.0348 (8)	0.0367 (8)	0.0064 (6)	0.0035 (6)	−0.0037 (7)
C11	0.0289 (7)	0.0380 (8)	0.0294 (7)	0.0036 (7)	0.0000 (6)	−0.0026 (6)
C12	0.0258 (7)	0.0393 (8)	0.0277 (7)	0.0007 (6)	−0.0042 (6)	−0.0005 (6)
C13	0.0385 (8)	0.0403 (9)	0.0384 (8)	0.0007 (7)	0.0038 (7)	−0.0042 (7)
C14	0.0401 (9)	0.0569 (11)	0.0420 (9)	0.0041 (8)	0.0081 (7)	−0.0113 (8)
C15	0.0338 (8)	0.0683 (11)	0.0392 (9)	−0.0040 (9)	0.0088 (7)	0.0015 (8)
C16	0.0334 (8)	0.0469 (9)	0.0463 (9)	−0.0051 (8)	−0.0005 (7)	0.0083 (8)
C17	0.0273 (7)	0.0386 (8)	0.0368 (8)	0.0036 (7)	−0.0016 (6)	0.0006 (7)
C20	0.0351 (8)	0.0450 (9)	0.0535 (10)	−0.0043 (7)	−0.0060 (8)	−0.0080 (8)
C18	0.0429 (9)	0.0710 (12)	0.0278 (7)	0.0021 (9)	0.0022 (7)	0.0029 (8)
O1	0.0494 (7)	0.0350 (6)	0.0568 (7)	0.0033 (5)	0.0149 (6)	0.0062 (5)

### Geometric parameters (Å, °)

**Table:**

C8A—C8	1.5153 (19)	C1—H1B	0.9700
C8A—C1	1.528 (2)	C1—H1A	0.9700
C8A—C4A	1.5439 (19)	C9—O1	1.2197 (17)
C8A—H1	0.9800	C9—C10	1.478 (2)
C8—C7	1.340 (2)	C10—C11	1.324 (2)
C8—C18	1.501 (2)	C10—H10	0.9300
C7—C6	1.493 (2)	C11—C12	1.4765 (19)
C7—H7	0.9300	C11—H11	0.9300
C6—C5	1.526 (2)	C12—C17	1.385 (2)
C6—H6A	0.9700	C12—C13	1.393 (2)
C6—H6B	0.9700	C13—C14	1.382 (2)
C5—C4A	1.527 (2)	C13—H13	0.9300
C5—H5A	0.9700	C14—C15	1.381 (3)
C5—H5B	0.9700	C14—H14	0.9300
C4A—C20	1.529 (2)	C15—C16	1.376 (2)
C4A—C4	1.537 (2)	C15—H15	0.9300
C4—C3	1.525 (2)	C16—C17	1.383 (2)
C4—H4B	0.9700	C16—H16	0.9300
C4—H4A	0.9700	C17—H17	0.9300
C3—C2	1.542 (2)	C20—H20A	0.9600
C3—H3A	0.9700	C20—H20B	0.9600
C3—H3B	0.9700	C20—H20C	0.9600
C2—C9	1.519 (2)	C18—H18C	0.9600
C2—C1	1.5304 (18)	C18—H18A	0.9600
C2—H2	0.9800	C18—H18B	0.9600
			
C8—C8A—C1	115.22 (12)	C3—C2—H2	108.4
C8—C8A—C4A	111.80 (11)	C8A—C1—C2	111.42 (11)
C1—C8A—C4A	112.03 (11)	C8A—C1—H1B	109.3
C8—C8A—H1	105.6	C2—C1—H1B	109.3
C1—C8A—H1	105.6	C8A—C1—H1A	109.3
C4A—C8A—H1	105.6	C2—C1—H1A	109.3
C7—C8—C18	120.41 (14)	H1B—C1—H1A	108.0
C7—C8—C8A	120.57 (14)	O1—C9—C10	118.67 (13)
C18—C8—C8A	118.96 (13)	O1—C9—C2	120.85 (13)
C8—C7—C6	125.23 (14)	C10—C9—C2	120.39 (12)
C8—C7—H7	117.4	C11—C10—C9	125.69 (13)
C6—C7—H7	117.4	C11—C10—H10	117.2
C7—C6—C5	112.24 (13)	C9—C10—H10	117.2
C7—C6—H6A	109.2	C10—C11—C12	126.55 (13)
C5—C6—H6A	109.2	C10—C11—H11	116.7
C7—C6—H6B	109.2	C12—C11—H11	116.7
C5—C6—H6B	109.2	C17—C12—C13	118.51 (14)
H6A—C6—H6B	107.9	C17—C12—C11	117.97 (13)
C6—C5—C4A	112.47 (13)	C13—C12—C11	123.50 (14)
C6—C5—H5A	109.1	C14—C13—C12	120.31 (16)
C4A—C5—H5A	109.1	C14—C13—H13	119.8
C6—C5—H5B	109.1	C12—C13—H13	119.8
C4A—C5—H5B	109.1	C15—C14—C13	120.45 (16)
H5A—C5—H5B	107.8	C15—C14—H14	119.8
C5—C4A—C20	109.37 (12)	C13—C14—H14	119.8
C5—C4A—C4	109.61 (12)	C16—C15—C14	119.72 (15)
C20—C4A—C4	110.34 (13)	C16—C15—H15	120.1
C5—C4A—C8A	107.89 (12)	C14—C15—H15	120.1
C20—C4A—C8A	111.77 (12)	C15—C16—C17	120.01 (16)
C4—C4A—C8A	107.80 (11)	C15—C16—H16	120.0
C3—C4—C4A	113.16 (13)	C17—C16—H16	120.0
C3—C4—H4B	108.9	C16—C17—C12	121.01 (15)
C4A—C4—H4B	108.9	C16—C17—H17	119.5
C3—C4—H4A	108.9	C12—C17—H17	119.5
C4A—C4—H4A	108.9	C4A—C20—H20A	109.5
H4B—C4—H4A	107.8	C4A—C20—H20B	109.5
C4—C3—C2	111.86 (13)	H20A—C20—H20B	109.5
C4—C3—H3A	109.2	C4A—C20—H20C	109.5
C2—C3—H3A	109.2	H20A—C20—H20C	109.5
C4—C3—H3B	109.2	H20B—C20—H20C	109.5
C2—C3—H3B	109.2	C8—C18—H18C	109.5
H3A—C3—H3B	107.9	C8—C18—H18A	109.5
C9—C2—C1	111.70 (12)	H18C—C18—H18A	109.5
C9—C2—C3	108.19 (12)	C8—C18—H18B	109.5
C1—C2—C3	111.58 (12)	H18C—C18—H18B	109.5
C9—C2—H2	108.4	H18A—C18—H18B	109.5
C1—C2—H2	108.4		
